# Health-related quality of life of children with first onset steroid-sensitive nephrotic syndrome

**DOI:** 10.1007/s00431-023-05135-5

**Published:** 2023-08-07

**Authors:** Floor Veltkamp, Lorynn Teela, Michiel A. J. Luijten, Hedy A. van Oers, Elske M. Mak-Nienhuis, Lotte Haverman, Antonia H. M. Bouts, on behalf of the LEARNS consortium, Abdul Adeel, Anna Bael, Antonia H. M. Bouts, Nynke H. Buter, Hans van der Deure, Eiske Dorresteijn, Sandrine Florquin, Valentina Gracchi, Flore Horuz, Francis Kloosterman-Eijgenraam, Elena Levtchenko, Elske M. Mak-Nienhuis, Ron A. A. Mathôt, Floor Oversteege, Saskia de Pont, Roos W. G. van Rooij-Kouwenhoven, Michiel F. Schreuder, Rixt Schriemer, Paul Vos, Johan Vande Walle, Joanna A. E. van Wijk

**Affiliations:** 1 Amsterdam University Medical Centers location University of Amsterdam, Emma Children’s Hospital, Department of Pediatric Nephrology, Meibergdreef 9, Post box 22660, 1100 DD Amsterdam, the Netherlands; 2 Amsterdam Reproduction and Development, Child Development, Amsterdam, The Netherlands; 3 Amsterdam University Medical Centers, location University of Amsterdam, Emma Children’s Hospital, Child and Adolescent Psychiatry & Psychosocial Care, Meibergdreef 9, Amsterdam, The Netherlands; 4 Amsterdam Public Health, Mental Health, Amsterdam, the Netherlands

**Keywords:** Nephrotic syndrome, Pediatric, Quality of life, Psychosocial functioning

## Abstract

This study assessed HRQoL and emotional and behavioral difficulties (EBD) and associated variables in children with first onset SSNS. While relapsing steroid-sensitive nephrotic syndrome (SSNS) in children is associated with lower health-related quality of life (HRQoL), little is known about first onset. Four weeks after onset, children (2–16 years) and/or their parents who participated in a randomized placebo-controlled trial, completed the Pediatric Quality of Life Inventory 4.0 (PedsQL) and Strengths and Difficulties Questionnaire (SDQ) to measure HRQoL and EBD, respectively. Total and subscale scores and the proportion of children with impaired HRQoL (> 1 SD below the mean of the reference group) or SDQ clinical scores (< 10^th^ and > 90^th^ percentile) were compared to the Dutch general population (reference group). Regression analyses were used to identify associated variables. Compared to the reference group, children 8–18 years reported significantly lower total HRQoL, and physical and emotional functioning. A large proportion (> 45%) of these children had impaired HRQoL. There were no differences in HRQoL between children 2–7 years and the reference group, except for higher scores on social functioning (5–7 years). Similar proportions of SSNS and reference children scored within the clinical range of SDQ subscales. Age, sex, and steroid side-effects were negatively associated with HRQol and/or EBD.

*Conclusion*: This study showed that HRQoL and EBD are affected in children of different ages with first onset SSNS. This calls for more awareness from healthcare providers and routinely monitoring of HRQoL and EBD in daily clinical care to prevent worsening of symptoms.

*Clinical trial registry*: Netherlands Trial Register (https://trialsearch.who.int/; NTR7013), date of registration: 02 June 2018.

**Table:** 

**What is Known:** *• Health-related quality of life (HRQoL) is lower and emotional and behavioral difficulties (EBD) is more affected in children with frequently-relapsing and steroid-dependent nephrotic syndrome.*	
**What is New:** *• HRQoL and EBD are affected in children with first onset steroid-sensitive nephrotic syndrome compared to a reference group of the Dutch general population.* *• To what extent HRQoL and EBD are affected depends on the age of the patient.*

**Supplementary Information:**

The online version contains supplementary material available at 10.1007/s00431-023-05135-5.

## Introduction

Idiopathic nephrotic syndrome (INS) is a rare glomerular disease in children that is characterized by the triad of profound edema, proteinuria, and hypoalbuminemia [1]. The first episode of INS is treated with a long course of high-dose corticosteroids [2–5], often leading to substantial side-effects, including Cushing symptoms, binge eating, hypertension, mood swings, and changed behavior [6]. Despite the good initial response – more than 85% of INS patients achieve complete remission (steroid-sensitive nephrotic syndrome (SSNS)) within 4 weeks of prednisolone – the risk for relapse is high: around 80% of children experience at least one relapse within 2 years after first onset [7–9]. Half of these patients experience multiple relapses per year (frequently relapsing nephrotic syndrome (FRNS)) or relapse during or shortly after steroid treatment (steroid-dependent nephrotic syndrome (SDNS)) [5].

Health-related quality of life (HRQoL) is the perception of one’s position in life when put in terms of physical symptoms, functional status, and disease impact on psychological and social functioning [10, 11], whereas psychosocial functioning is a person's ability to perform daily tasks and to interact with others and with society in a mutually satisfying manner [12, 13]. To measure HRQoL and psychosocial functioning focusing on emotional and behavioral difficulties (EBD), patient-reported outcome measures (PROMs, i.e. questionnaires) are used.

The relapsing–remitting behavior of SSNS and the marked side-effects of treatment negatively impact HRQoL in children with SSNS. Previous studies have shown that HRQoL in INS patients is lower compared to their peers [14–17], but better than in children with other chronic illnesses [18, 19]. However, these studies focused on relapsing SSNS (SDNS and/or FRNS). Studies about HRQoL and EBD in the first weeks of confirmed SSNS are lacking. Therefore, this study aims to determine the HRQoL and EBD and their association with sociodemographic and clinical variables of children between 2 and 16 years of age four weeks after onset of SSNS.

## Methods

### Study design

This is a prospective, cross-sectional, multicenter, observational study that is part of the LEARNS randomized, placebo-controlled trial studying the efficacy and safety of alternate day levamisole added to standard corticosteroid therapy to prevent relapses of SSNS [20]. Children were followed until 2 years after first onset. PROMs were collected at randomization before start of study medication (week 4), after discontinuation of study medication (week 28), at the primary endpoint (year 1), and at the end of the study (year 2). For this study, only the PROMs completed at week 4 were used for analysis to evaluate the effect of diagnosis and initial treatment at first onset on HRQoL and EBD.

PROMs were completed online using the KLIK PROM portal (www.hetklikt.nu); designed for monitoring patient outcomes in daily clinical practice and research [21]. After online registration, PROMs had to be completed within 14 days. After 7 days, participants received a reminder.

### Study participants

Children (aged 2–16 years) with first onset SSNS were screened for eligibility. SSNS was defined as the presence of proteinuria (urinary protein-creatinine ratio (uPCR) > 200 mg/mmol creatinine), hypoalbuminemia (serum albumin < 25 g/L), and edema achieving complete remission (uPCR < 50 mg/mmol) within 4 weeks of standard prednisolone therapy [5, 20]. All subjects received an 18-week prednisolone tapering schedule [2].

### Data collection and measures

Clinical data of the children were prospectively collected by trained clinicians using a standardized electronic case report form (Castor EDC), including age at onset (years), sex (male/female), cumulative prednisolone dose (mg/m^2^), time-to-remission (days), medical history of the past 4 weeks, school absence in the past 4 weeks, the presence of steroid side-effects (pre-specified: Cushingoid features, mood changes, binge eating, behavioral problems, hypertension, weight gain, skin abnormalities, and other), and concomitant medication use. Behavioral problems included aggression, bad attitude, anger, impatience, or change in and/or other problematic behavior according to parents.

Parents completed a survey on sociodemographics (age, sex, family situation, marital status, education). For Belgian children, versions appropriate for language (Flemish or French) were available. Two PROMs (Pediatric Quality of Life Inventory and Strengths and Difficulties Questionnaire) with appropriate versions for different age groups, were used to measure HRQoL and EBD.

#### Pediatric quality of life inventory (PedsQL) generic scale, version 4.0

The Dutch version of the PedsQL Generic Scale version 4.0 was used to measure HRQoL in children of different age categories (2–4 years, 5–7 years, 8–12 years, and 13–17 years) [22]. Children ≥ 8 years completed the PROM themselves (self-report). For children aged 2–7 years, we used proxy-report. The PedsQL consists of 21 (2–4 years) or 23 items (5–17 years) divided over four subscales: physical (n = 8), emotional (n = 5), social (n = 5), and school (n = 3/n = 5) functioning. Total HRQoL is the summary score of all subscales, while psychosocial functioning is that of emotional, social, and school functioning. Each item was scored on a 5-point Likert-scale. Items were reverse-scored and transformed to a 0–100 scale. Higher scores indicate better HRQoL. The PedsQL has a short completion time, and good feasibility, validity, and reliability [22, 23]. Reference data from the Dutch general population (n = 1286) are available and were stratified for key demographics, including age, sex, and education level, used as reference group [24, 25]. In our study, Cronbach’s alphas ranged between 0.64 and 0.93.

#### Strengths and difficulties questionnaire (SDQ)

The SDQ is a brief screening questionnaire developed for assessing EBD in children and adolescents [26]. Twenty-five items describe positive and negative attributes of children divided over 5 subscales (Emotional symptoms, Conduct problems, Hyperactivity-inattention, Peer problems, and Prosocial behavior). Items were scored on a 3-point scale and summed up to a 0–10 scale score. Higher scores on Prosocial behavior reflect strength, higher scores on all other scales reflect difficulties. The Total difficulties score is the sum of scales reflecting difficulties (range 0–40), the Internalizing problems score is the sum of Emotional symptoms and Conduct problems, and Externalizing problems is the sum of Hyperactivity-inattention and Peer problems (range 0–20). Scores > 90^th^ (for difficulties) or < 10^th^ (for Prosocial behavior) percentile are considered ‘clinical’ [26, 27]. The SDQ has both parent-reported (children 2–3 and 4–17 years old) and self-reported (11–17 years old) versions. The Dutch SDQ has acceptable to good psychometric properties [28]. Reference data for parent- and self-reported scores are available and were used as reference group [29, 30]. Due to low internal consistency within the reference data [29], scores for several subscales of the 2–3 and 4–5 years age groups were not presented. Cronbach’s alphas in our sample ranged between 0.32 and 0.94.

### Statistical analysis

Continuous data were presented as the mean ± standard deviation (SD) or median (range), according to distribution. Normal distribution was tested using histograms and QQ-pots. Discrete data were presented as frequencies and proportions (%).

Total and subscale PedsQL scores were calculated for each age category. Differences between children with SSNS (SSNS group) and reference group were tested by an independent t test. The proportion (%) of children with impaired HRQoL, based on a score of ≥ 1 SD below the mean of the reference group, were compared by Fisher’s exact test. Individual items scores were presented as proportions (%). For each SDQ (summary) subscale, scores were calculated per age group. SDQ scores of the SSNS and reference group were compared using a one-sample t test. To quantify the differences, effect sizes (Cohen’s *d*) were calculated with 95% confidence intervals (CIs). Effect size was calculated as the difference in mean scores between the SSNS and reference group divided by the pooled SD. Effect sizes of 0.2, 0.5, and 0.8 were considered small, moderate, and large, respectively [31]. For each subscale of the PedsQL and the SDQ, the Cronbach’s alpha (α) was calculated. Scores of a scale with an α < 0.50 were not presented.

Multiple linear regression models were used to identify associated variables with PedsQL and SDQ scores. First, the following variables were tested by univariate analyses: child’s age and sex, older age ate onset (above median), parent’s country of birth (Netherlands/Belgium versus other) and educational level (low, intermediate, or high), time-to-remission, late remission (above median), illness in the past 4 weeks, school absence in the past 4 weeks, > 1 week of illness, > 1 week of school absence, the presence of any and pre-specified steroid side-effects (if present in ≥ 25% of the children), and the use and number of medications. Variables with a *P* < 0.05 in at least one of the scales were included simultaneously in the multiple linear regression model. Correlation between variables was tested for multicollinearity. A correlation of > 0.8 was considered too high, which was not the case for any of the variables. To express the association between variables and outcome, standardized regression coefficients (β) were reported. For continuous variables, β were considered small if 0.1, medium if 0.3, and large if 0.5, while for dichotomous variables, β were considered small if 0.2, medium if 0.5, and large if 0.8 [31].

Data were analyzed using IBM SPSS Statistics for Windows [32] and R Studio [33, 34]. A *P* < 0.05 was considered statistically significant.

## Results

From April 25^th^, 2018 to December 10^th^, 2021, a total of 46 patients were included in the LEARNS study. Four weeks after first onset, 40 (87%) children and/or their parent(s) completed the PROMs. As a result of the design of the KLIK PROM portal (questions cannot be skipped), there were no missing data. Median age of the SSNS group was 6.3 (range 2.6–15.5) years. At least one side-effect of prednisolone was present in the majority of children (> 80%), binge eating (62.5%) and moon face (55.0%) being most prevalent. Table 1 shows the baseline characteristics.

**Table 1:** Baseline characteristics of children with SSNS at 4 weeks after first presentation

	**All ages** **(N = 40)**
**Country of residence, n (%)**	
Belgium	5 (12.5)
Netherlands	35 (87.5)
**Age (years), median (min, max)**	6.34 (2.6, 15.5)
**Male, n (%)**	25 (62.5)
**Parent born in the Netherlands/Belgium**	33 (82.5)
**Educational level of parent, n (%)**^**a**^	
Low	6 (15.0)
Intermediate	13 (32.5)
High	21 (52.5)
**Time to remission (days), median (min, max)**	10 (5, 30)
Late remission (> 10 days after start treatment), n (%)	20 (50.0)
**Prescribed prednisolone dose (mg/dose), mean (SD)**	51.0 (9.4)
**Cumulative prednisolone dose (mg), mean (SD)**	1590 (354)
**Cumulative prednisolone dose (mg/m**^**2**^**), mean (SD)**	1890 (667)
**Illness in past 4 weeks, n (%)**	11 (27.5)
Days of illness, median (min, max)^b^	2 (1, 10)
Illness for > 1 week, n (%)^b^	1 (2.5)
**School absence in past 4 weeks, n (%)**	14 (35.0)
Days of school absence, median (min, max)^b^	10 (1, 28)
School absence for > 1 week^b^	8 (20.0)
**Presence of steroid side-effects, n (%)**	34 (85.0)
Binge eating, n (%)	25 (62.5)
Moon face, n (%)	22 (55.0)
Behavioural changes, n (%)	17 (42.5)
Mood changes, n (%)	18 (45.0)
Weight gain, n (%)	12 (30.0)
**Use of concomitant medication, n (%)**	35 (87.5)
Number of used medications, mean (SD)	2.25 (1.1)
Vitamin D, n (%)	34 (85.0)
Proton pump inhibitors, n (%)	11 (27.5)
Antihypertensive medication, n (%)	4 (10.0)
Inhalation medication, n (%)	3 (7.5)
Antibiotics, n (%)	3 (7.5)
Allergy medication, n (%)	2 (5.0)
Other, n (%)	11 (27.5)

^a^Low: primary education, lower vocational education, lower or middle general secondary education; Intermediate: middle vocational education, higher secondary education, pre-university education; High: higher vocational education, university

^b^Proportion of children who were ill or missed school in the past 4 weeks

### Health-related quality of life

The results are shown in Table 2. No differences between the SSNS and reference group in age or sex were found in each age group. In children with SSNS aged 2–4 years, no significant differences in HRQoL scores were found, but moderate effect sizes were found in physical (*d* = 0.69, 95% CI 0.04–1.06) and school functioning (*d* = 0.73, 95% CI 0.22–1.24). Social functioning was significantly higher in children with SSNS aged 5–7 years compared to reference group (95.8 ± 6.7 vs. 86.4 ± 16.7, *P* < 0.001), with a moderate effect size *d* -0.57 (95% CI -1.13- -0.01). The HRQoL scores of children with SNSS aged 8–17 years were significantly lower compared to reference group on total (75.4 ± 14.4 vs. 84.9 ± 12.6, *P* = 0.013), physical (73.0 ± 20.8 vs. 91.6 ± 12.4, *P* = 0.014) and emotional functioning (63.6 ± 23.5 vs. 79.3 ± 18.4, *P* = 0.005) with moderate to large effect sizes (*d* ≥ 0.75).

**Table 2:** Health-related quality of life scores (mean ± SD) of the PedsQL for all age groups compared to the reference group. Higher scores mean better HRQoL. Effect sizes of 0.2, 0.5, and 0.8 are considered small, moderate, and large, respectively. *P*-values < 0.05 and effect sizes > 0.8 are shown in bold

		**2–4 years** **(proxy-report)**				**5–7 years** **(proxy-report)**				**8–17 years** **(self-report)**		
	**LEARNS** **(N = 16)**	**Reference** **(N = 293)**	***P***		**LEARNS** **(N = 13)**	**Reference** **(N = 274)**	***P***		**LEARNS** **(N = 11)**	**Reference** **(N = 966)**	***P***	
**Age, years, mean ± SD**	3.8 ± 0.8	3.5 ± 0.9	0.09		6. 8 ± 0.8	6.5 ± 0.9	0.28		12.0 ± 2.3	13.1 ± 2.8	0.13	
**Male, n (%)**	9 (56.2)	157 (53.6)	1		8 (61.5)	152 (55.5)	0.78		8 (72.7)	496 (51.3)	0.23	

	**LEARNS** **(N = 16)**	**Reference** **(N = 293)**	***P***	***d* (95% CI)**	**LEARNS** **(N = 13)**	**Reference** **(N = 274)**	***P***	***d* (95% CI)**	**LEARNS** **(N = 11)**	**Reference** **(N = 966)**	***P***	***d* (95% CI)**
**Total score**	82.8 ± 14.4	88.4 ± 10.0	0.15	0.55 (0.04–1.06)	87.0 ± 8.05	86.0 ± 11.6	0.78	-0.09 (-0.65–0.47)	75.4 ± 14.4	84.9 ± 12.6	**0.013**	0.75 (0.15–1.35)
**Physical functioning**	83.8 ± 17.7	91.8 ± 11.2	0.09	0.69 (0.18–1.20)	88.5 ± 8.9	91.1 ± 12.6	0.47	0.21 (-0.35–0.77)	73.0 ± 20.8	91.6 ± 12.4	**0.014**	**1.49 (0.89–2.09)**
**Emotional functioning**	75.9 ± 18.2	78.4 ± 14.7	0.51	0.17 (-0.33–0.67)	73.1 ± 14.8	77.9 ± 16.5	0.30	0.29 (-0.27–0.85)	63.6 ± 23.5	79.3 ± 18.4	**0.005**	**0.85 (0.25–1.45)**
**Social functioning**	86.9 ± 14.9	90.1 ± 13.7	0.36	0.23 (-0.27–0.73)	95.8 ± 6.7	86.4 ± 16.7	** < 0.001**	-0.57 (-1.13- -0.01)	90.5 ± 9.6	84.4 ± 16.9	0.06	-0.36 (-0.95–0.23)
**School functioning**	84.9 ± 18.1	93.7 ± 11.7	0.07	0.73 (0.22–1.24)	89.6 ± 11.1	85.8 ± 15.4	0.38	-0.25 (-0.81–0.31)	75.9 ± 17.6	80.4 ± 16.8	0.38	0.27 (-0.32–0.86)
**Psychosocial functioning**	82.2 ± 13.4	86.2 ± 11.2	0.17	0.35 (-0.15–0.85)	86.2 ± 8.3	83.4 ± 13.7	0.47	-0.21 (-0.77–0.35)	76.7 ± 13.4	81.4 ± 14.7	0.29	0.32 (-0.27–0.91)

*CI* confidence interval, *HRQoL* health-related quality of life, *PedsQL* Pediatric Quality of Life Inventory 4.0, *SD* standard deviation

**Fig. 1:**
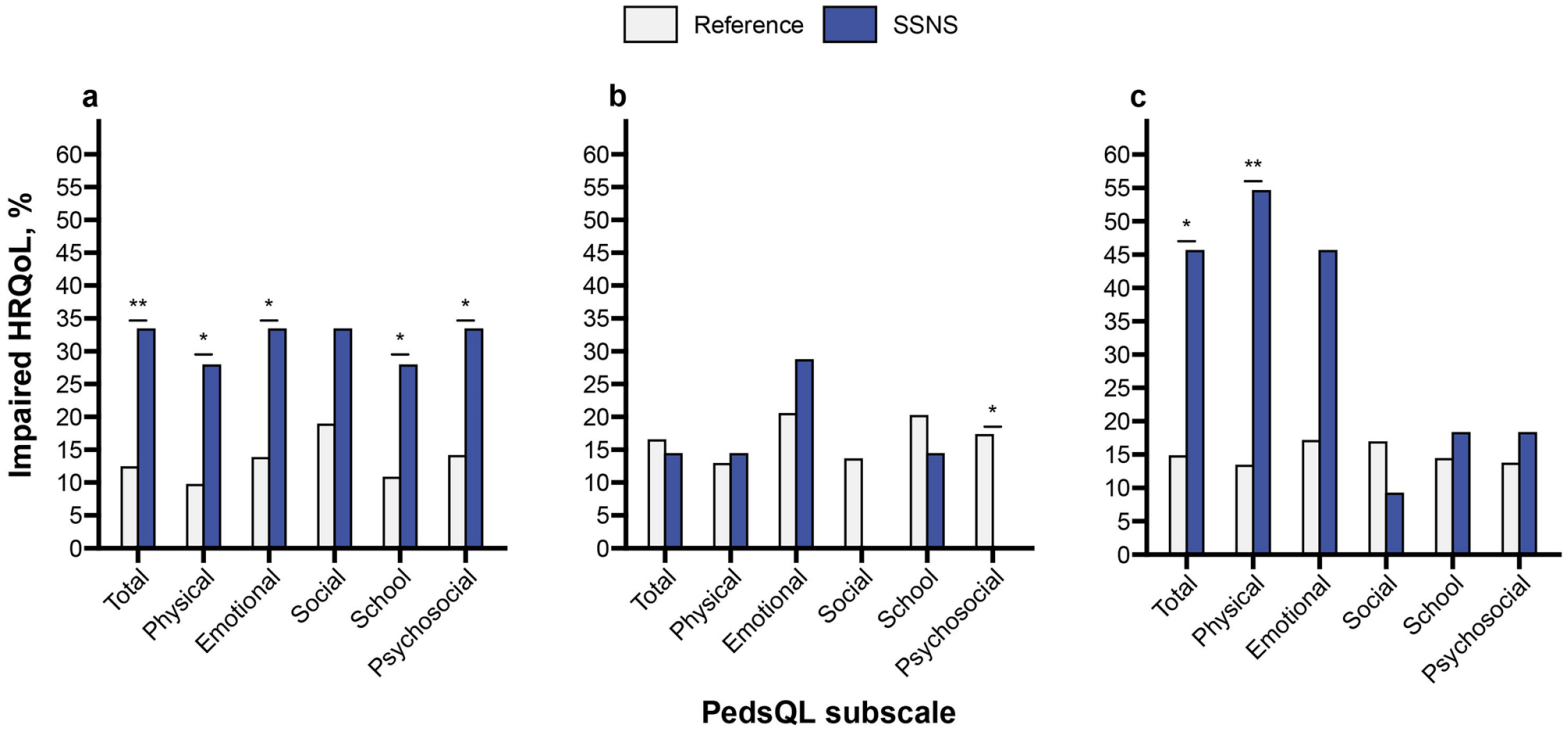
Proportions (%) of children with impaired HRQoL based a score of ≥ 1SD below the mean of the Dutch norm population for ages **a** 2–4 years (proxy-report), **b** 5–7 years (proxy-report), and **c** 8–17 years (self-report). * = P < 0.05; ** = P < 0.01

Compared to the reference group, HRQoL was more often impaired in children aged 2–4 years on all subscales except social functioning (Fig. 1a). In children aged 5–7 years, psychosocial functioning was significantly less often impaired (0% vs. 17%) (Fig. 1b). A significantly larger proportion of children aged 8–17 years reported impaired total (46% vs. 15%), physical (55% vs. 13%) and emotional functioning (46% vs. 17%) compared to the reference group (Fig. 1c). On the individual items of the PedsQL, older children with SNSS reported to experience more pain, lower energy levels, trouble sleeping, feeling angry, and worrying than their peers (Online Resource I – Table S1c).

### Emotional and behavioural difficulties

**Fig. 2:**
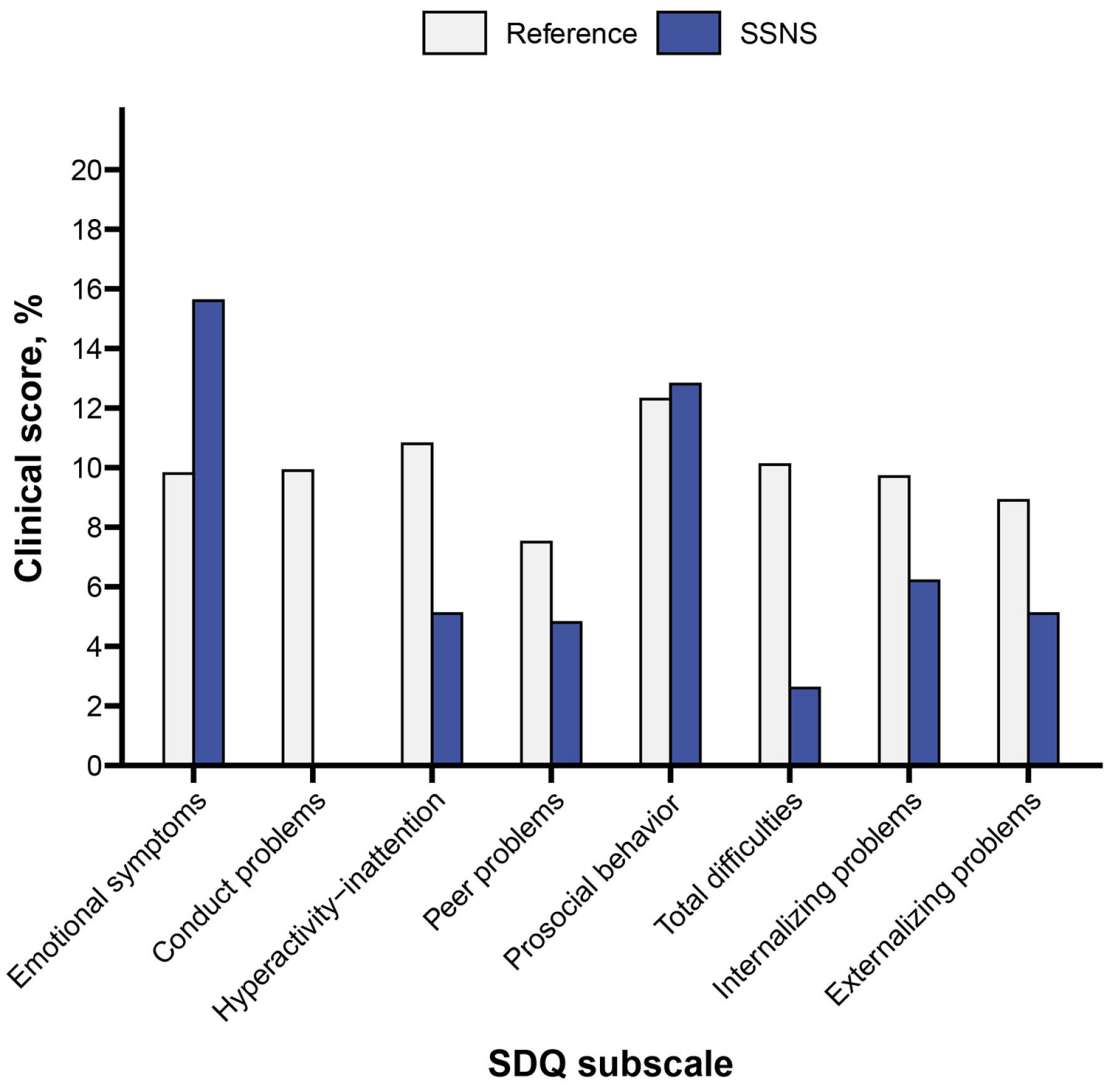
Proportions (%) of children with clinical scores, defined as a score < 10^th^ (strengths) or > 90^th^ percentile (difficulties) of the mean score of Dutch norm population for all parent-reported age groups (2–17 years) combined (N = 39). None of the SSNS children had clinical scores for Conduct problems

The results of the SDQ are presented in Online Resource I – Table S2. Compared to the reference group, more children with SSNS (2–17 years, parent-reported) scored within ‘clinical’ range (15.6 vs. 9.8%) of Emotional functioning (Fig. 2), but this difference was not significant. However, significantly lower mean scores on Conduct, Peer and Internalizing problems, and Total difficulties were reported by parents of 6–11-year-olds with SSNS, indicating fewer difficulties than their peers. They also scored higher on Prosocial behavior, indicating more strengths. Parents of children with SSNS aged 12–17 years (also proxy-report) indicated that their child had more Emotional symptoms, but better Prosocial behavior than the reference group (Online Resource I – Table S2).

### Associated variables with HRQoL and psychosocial functioning

Age and time-to-remission were included in the multiple regression analysis of the PedsQL, while age, male sex, school absence, binge eating and behavioral problems were included in multiple regression analysis of the SDQ (Online Resource I – Table S3). Multiple regression identified that higher age (β = -0.34) was negatively associated with the emotional functioning subscale of the PedsQL (Table 3a). Higher age was also associated with better Prosocial behavior scale on the SDQ (β = 0.48). Boys reported more Peer problems on the SDQ (β = 0.32) than girls. When behavioral problems from steroids were present, parents reported higher Hyperactivity-inattention (β = -0.34) and Externalizing problems (β = 0.36) scores, while binge eating was associated with less Emotional symptoms on the SDQ (β = -0.34) (Table 3b).

**Table 3:** Results from the multiple linear regression analyses of **a**. the PedsQL and **b**. SDQ questionnaires. The effect of the included variables is expressed as standardized regression coefficients (β)

**a. PedsQL**	**Total** **score**	**Physical** **functioning**	**Emotional functioning**	**Social** **functioning**	**School** **functioning**	**Psychosocial functioning**
**Age child**	-0.29	-0.30	**-0.34**^a^	0.08	-0.31	-0.28
**Time-to-remission**	-0.13	-0.11	-0.21	-0.04	-0.07	0.09

**b. SDQ**	**Emotional symptoms**	**Conduct problems**	**Hyperactivity-inattention**	**Peer** **problems**	**Prosocial behaviour**	**Total difficulties**	**Internalizing problems**	**Externalizing problems**
**Age child**	0.24	0.06	-0.01	-0.25	**0.48**^b^	-0.03	0.03	-0.01
**Sex child**	-0.05	0.30	0.01	**0.32**^a^	-0.18	0.15	0.14	0.10
**School absence (days)**	-0.11	-0.23	0.00	-0.11	0.21	-0.13	-0.14	-0.07
**Behavioral problems**	-0.04	0.29	**0.34**^a^	-0.19	0.19	0.20	-0.08	**0.36**^a^
**Binge eating**	**-0.34**^a^	-0.01	0.07	0.11	-0.07	-0.07	-0.19	0.06

^a^*P* < 0.05; ^b^*P* < 0.001

## Discussion

The results of this study show that at first onset of SSNS, HRQoL and EBD are already affected in children of different ages (2–16 years) compared to a reference group of the Dutch general population. However, not all age groups were equally affected. The effect was more profound in older children (≥ 8 years) than in younger children, with children aged 5–7 years scoring even better on social functioning.

Total HRQOL, physical and emotional functioning were significantly affected in children aged 8–17 years. Physical functioning can be impacted by limitations in daily activities, while mood disturbances – whether caused by steroids – can affect emotional functioning. More specifically, on the individual items, children with SNSS indicated that they experienced more pain, lower energy levels, and trouble sleeping (physical functioning), and feeling angry and worrying (emotional functioning) than their peers. Older children may be more aware of their condition than younger children, who scored comparable to their peers. Social functioning was not affected in any age group. Children 5–7 years scored even better on social functioning than reference and reported having fewer problems with getting along with peers or making friends. This could be explained by the fact that they may fare well by the attention received from family, friends, and schoolmates. Although children in our study have only been ill for four weeks, this is in line with previous studies, in which social functioning was also unaffected in children with a chronic illness [25, 35, 36].

This is the first study in children with first onset SSNS and in whom disease is in remission. Our findings are in line with those of Selewski et al. who reported that HRQoL – measured by PedsQL – in American children aged 8–17 years old with incident INS (i.e. first onset within 14 days of therapy) was worse than peers on physical, social, and total functioning [37]. To date, this had been the only HRQoL study in first onset SSNS. However, that study was conducted in children with active INS of 8 years or older only. In our study, children of all ages were studied using both proxy- and self-reported outcomes. Since SSNS predominantly affects children under the age of 6 years, our results fill this knowledge gap. Moreover, in our study, each child was in remission at PROM completion, but still receiving high doses of prednisolone. The poor scores on physical, social and total functioning could therefore be an effect of steroid treatment rather than of the disease itself.

Previous studies have found an association between the use and cumulative dose of steroids and HRQoL [38]. In children with relapsing SSNS, there is a wide variation in cumulative steroid dose and the use of steroid-sparing agents, which have been identified as independent predictors for negative outcome [39]. Disease duration [19, 37], the number of relapses [19], and disease severity [15, 16] have been identified as factors that negatively impact HRQoL in children with SSNS. As children in our study were at first onset having received similar doses of prednisolone, these factors could not be explored. Yet, as these children will be followed for two years after, systematically collected longitudinal HRQoL data will be available later. With this study, we provided a baseline score for children who are in remission.

At four weeks after onset, children are at their peak of steroid side-effects. While toxicity still continues after four weeks, study subjects were switched to alternate day prednisolone (60 mg/m^2^) at week 4 according to study protocol [2, 20], greatly relieving the steroid burden. We found that the presence of behavioral problems as reported by parents to the clinician were associated with more Hyperactivity-inattention and Externalizing problems. It is known that problematic externalizing behavior, such as aggression, violence, and restlessness, is more likely to be reported as it is visible behavior compared to internalizing behavior (anxiousness, depression, and refraining from social activities) which is an internal sense for the child. Surprisingly, binge eating was associated with fewer Emotional symptoms.

As Selewski et al. [37] demonstrated, HRQoL at first onset (incident INS) is worse than in the general population, but better than later in the disease course of SSNS (prevalent INS). Knowing more about the evolution of HRQoL and EBD over different courses of SSNS, could contribute to more patient-centered care. Monitoring and discussing HRQoL in daily clinical practice may aid clinicians with timely identifying lower HRQOL or more EBD and provide tailored interventions to prevent worsening of and/or improve symptoms. The KLIK PROM portal is a user-friendly and effective way to do so [40, 41].

This study was limited by small sample sizes per age group, which is secondary to the low incidence of SSNS. Due to a lack of power, differences that may be clinically relevant could have been missed. Therefore, we calculated effect sizes (Cohen’s *d*) which indeed showed moderate effects for the limited number of younger children. Also, a considerable number of variables have been tested for an association with outcome, increasing the chance of finding a false-positive result. Also, we acknowledge that HRQoL at 4 weeks after first onset may not be representative for the months following. For this reason, a long-term follow-up is currently ongoing. Last, HRQoL was only measured in children who participated in the LEARNS study, which may have biased the results and positively skewed the results.

On the other hand, this study is strengthened by the fact that this is the first study in children with first onset SSNS, adding new insights into the well-being of these children. The study consisted of a homogeneous population that spanned a wide age range (2–16 years), now including children in which SSNS is more incident. Furthermore, a high response rate was achieved (86%), which can be owed to the prospective study design, use of an online PROM portal, and a dedicated study team. Finally, recent Dutch reference data for both validated PedsQL and SDQ were available.

## Conclusions

Impaired HRQoL was more profound in older children with first onset SSNS, in whom considerable proportions of impaired physical, emotional and total functioning were observed. Furthermore, this study showed that HRQoL and EBD are already affected in the first weeks after onset. Therefore, systematically monitoring of HRQoL and EBD by the use of PROMs should be encouraged to improve patient-centered care and timely identify signs of impaired HRQoL and EBD in children with first onset SSNS. Collection of longitudinal data of the first 2 years after onset is still ongoing and will provide additional insight into predictors of HRQoL outcomes eventually.

### Supplementary Information

Below is the link to the electronic supplementary material.

Supplementary file1 (PDF 770 KB)

## Data Availability

Deidentified individual participant data (including data dictionaries) will be made available, in addition to study protocols, the statistical analysis plan, and the informed consent form. The data will be made available upon publication to researchers who provide a methodologically sound proposal for use in achieving the goals of the approved proposal. Proposals should be submitted to the corresponding author.

## Abbreviations (in alphabetical order)

CI: Confidence interval
EBD: Emotional and behavioral difficulties
FRNS: Frequently-relapsing nephrotic syndrome
HRQoL: Health-related quality of life
INS: Idiopathic nephrotic syndrome
PedsQL: Pediatric Quality of Life Inventory 4.0
PROM: Patient-reported outcome measure
SD: Standard deviation
SDNS: Steroid-dependent nephrotic syndrome
SDQ: Strengths and Difficulties Questionnaire
SSNS: Steroid-sensitive nephrotic syndrome
uPCR: Urinary protein-creatinine ratio
