# Supplementary material for: Health-related quality of life of children with first onset steroid-sensitive nephrotic syndrome
Source: Eur J Pediatr. 2023 Aug 7;182(10):4605–14. doi: 10.1007/s00431-023-05135-5 (PMC10587200; doi:10.1007/s00431-023-05135-5)
Supplement: Supplementary file 1 — Supplementary file1 (PDF 770 KB) [file 431_2023_5135_MOESM1_ESM.pdf]

Online Resource 1

to:

Health-related quality of life and emotional behavioral difficulties in children with first onset steroid-sensitive nephrotic syndrome

Floor Veltkamp[1,2], MD, Lorynn Teela[2,3], MSc, Michiel A.J. Luijten[2,3], MSc, Hedy A. van Oers[2,3,4], PhD, Elske M. Mak-Nienhuis[1], Lotte Haverman[2,3,4]†, PhD, and Antonia H.M. Bouts[1,2]†, MD PhD, on behalf of the LEARNS consortium‡.

† Contributed equally as co-senior authors.

‡ A complete list of study group members appears in the Acknowledgments.

[1] Amsterdam University Medical Centers location University of Amsterdam, Emma Children's Hospital, Department of Pediatric Nephrology, Meibergdreef 9, Amsterdam, the Netherlands.

[2] Amsterdam Reproduction and Development, Child development, Amsterdam, The Netherlands.

[3] Amsterdam UMC location University of Amsterdam, Emma Children's Hospital, Child and Adolescent Psychiatry & Psychosocial Care, Meibergdreef 9, Amsterdam, The Netherlands.

[4] Amsterdam Public Health, Mental health, Amsterdam, the Netherlands.

Corresponding author: Antonia H.M. Bouts, MD PhD, Amsterdam University Medical Centers, University of Amsterdam, Emma Children's Hospital, Department of Pediatric Nephrology, Post box 22660, 1100 DD, Amsterdam, the Netherlands.

a.h.bouts@amsterdamumc.nl

Table S1 Item scores of the PedsQL questionnaire per age category, shown as proportions (%)

a. PedsQL 2-4 years

		Almost always	Often	Some-times	Almost never	Never
Physical functioning						
Hard to walk more than one block	LEARNS (n=16)	0	0	13	6	81
	Norm (n= 293)	1.4	0	2.4	3.4	93
Hard to run	LEARNS (n=16)	0	6	13	6	75
	Norm (n= 293)	1.7	0.3	2.4	6.1	89
Hard to do sports or exercises	LEARNS (n=16)	0	13	6	13	69
	Norm (n= 293)	1.4	0.7	2	5.1	91
Hard to lift something heavy	LEARNS (n=16)	0	0	6	13	81
	Norm (n= 293)	0.7	0.3	4.8	9.2	85
Hard to take a bath or shower	LEARNS (n=16)	0	6	0	13	81
	Norm (n= 293)	1	1.4	3.1	5.5	89
Hard to do chores / clean up toys	LEARNS (n=16)	0	6	6	19	69
	Norm (n= 293)	0.7	3.4	18	20	57
Hurt or ache	LEARNS (n=16)	0	0	50	19	31
	Norm (n= 293)	0	1.4	13	26	60
Low energy	LEARNS (n=16)	0	19	38	19	25
	Norm (n= 293)	0.3	0.7	9.9	20	69
Emotional functioning						
Feel afraid or scared	LEARNS (n=16)	0	6	31	6	56
	Norm (n= 293)	0	1.7	24	37	37
Feel sad or blue	LEARNS (n=16)	0	0	50	19	31
	Norm (n= 293)	0	0.3	15	38	46
Feel angry	LEARNS (n=16)	0	0	50	38	13
	Norm (n= 293)	0	4.4	42	33	20
Trouble sleeping	LEARNS (n=16)	0	6	6	31	56
	Norm (n= 293)	0.7	4.4	18	32	45
Worry about what will happen	LEARNS (n=16)	6	0	19	13	63
	Norm (n= 293)	0	1.7	9.2	34	55
Social functioning						
Trouble getting along with peers	LEARNS (n=16)	6	6	19	13	56
	Norm (n= 293)	1	2.7	8.9	28	59
Other kids not wanting to be friends	LEARNS (n=16)	0	0	0	19	81
	Norm (n= 293)	0.7	0.3	4.1	17	78
Teased	LEARNS (n=16)	0	0	0	13	88
	Norm (n= 293)	0	0	2.7	14	83
Doing things other peers do	LEARNS (n=16)	0	6	19	19	56
	Norm (n= 293)	1.4	2	7.2	20	69
Hard to keep up when play with others	LEARNS (n=16)	0	6	13	19	63
	Norm (n= 293)	2.7	3.1	4.8	16	73
School functioning						
Trouble keeping up with activities	LEARNS (n=16)	0	0	13	13	75
	Norm* (n= 293)	3.3	1.6	4.1	11	80
Miss school – not well	LEARNS (n=16)	0	13	6	25	56
	Norm* (n= 293)	0.4	0.4	3.3	13	83
Miss school – doctor appointment	LEARNS (n=16)	0	0	25	19	56
	Norm* (n= 293)	0	0.8	2.5	9.1	88

*Data missing for 50 respondents.

b. PedsQL 5-7 years

		Almost always	Often	Some-times	Almost never	Never
**Physical functioning**						
Hard to walk more than one block	LEARNS (n=13)	0	0	0	0	100
	Norm (n= 274)	1.1	0	1.8	2.9	94
Hard to run	LEARNS (n=13)	0	0	0	23	77
	Norm (n= 274)	1.5	2.2	2.6	2.9	91
Hard to do sports or exercises	LEARNS (n=13)	0	0	31	8	62
	Norm (n= 274)	1.1	1.5	4.7	4.4	88
Hard to lift something heavy	LEARNS (n=13)	0	0	8	15	77
	Norm (n= 274)	0.4	0.7	2.9	4.7	91
Hard to take a bath or shower	LEARNS (n=13)	0	0	0	8	92
	Norm (n= 274)	2.9	4.7	4.4	7.7	80
Hard to do chores around the house	LEARNS (n=13)	0	0	0	15	85
	Norm (n= 274)	1.1	5.1	11	17	66
Hurt or ache	LEARNS (n=13)	0	0	38	23	38
	Norm (n= 274)	0.4	1.5	15	34	49
Low energy	LEARNS (n=13)	0	8	38	23	31
	Norm (n= 274)	0	1.8	11	19	68
**Emotional functioning**						
Feel afraid or scared	LEARNS (n=13)	0	0	15	38	46
	Norm (n= 274)	0	4.7	23	34	38
Feel sad or blue	LEARNS (n=13)	0	8	38	23	31
	Norm (n= 274)	0	2.6	19	39	40
Feel angry	LEARNS (n=13)	0	31	38	15	15
	Norm (n= 274)	0	8.4	41	33	18
Trouble sleeping	LEARNS (n=13)	0	8	31	23	38
	Norm (n= 274)	1.5	4.4	15	26	53
Worry about what will happen	LEARNS (n=13)	0	0	15	23	62
	Norm (n= 274)	0.4	2.6	9.1	20	68
**Social functioning**						
Trouble getting along with peers	LEARNS (n=13)	0	0	8	31	62
	Norm (n= 274)	5.1	3.3	9.5	25	57
Other kids not wanting to be friends	LEARNS (n=13)	0	0	0	0	100
	Norm (n= 274)	0.4	2.9	7.3	26	64
Teased	LEARNS (n=13)	0	0	0	0	100
	Norm (n= 274)	0	0.7	8.4	28	63
Doing thing other peers do	LEARNS (n=13)	0	0	0	8	92
	Norm (n= 274)	0.7	2.2	8	22	68
Hard to keep up when play with others	LEARNS (n=13)	0	0	8	15	77
	Norm (n= 274)	2.6	4	5.1	22	67
**School functioning**						
Focus	LEARNS (n=13)	0	0	8	15	77
	Norm (n= 274)	2.2	7.3	21	22	48
Forgetting things	LEARNS (n=13)	0	0	0	31	69
	Norm (n= 274)	0	5.8	13	24	57
Trouble keeping up with schoolwork	LEARNS (n=13)	0	0	0	23	77
	Norm (n= 274)	2.6	4.4	11	19	63
Miss school – not well	LEARNS (n=13)	0	8	0	15	77
	Norm (n= 274)	0	0.7	5.5	15	79
Miss school – doctor appointment	LEARNS (n=13)	0	0	31	23	46
	Norm (n= 274)	0	1.1	3.6	19	76

c. PedsQL 8-17 years

		Almost always	Often	Some-times	Almost never	Never
Physical functioning						
Hard to walk more than one block	LEARNS (n=11)	0	9	18	9	64
	Norm (n=964)	0.4	0.3	2.3	6	91
Hard to run	LEARNS (n=11)	0	9	27	9	55
	Norm (n=964)	0.6	2.5	5.1	11.5	80
Hard to do sports or exercises	LEARNS (n=11)	0	18	36	27	18
	Norm (n=964)	0.8	1.9	7	11.2	19.1
Hard to lift something heavy	LEARNS (n=11)	0	18	27	18	36
	Norm (n=964)	0.7	0.9	6.8	17.4	74.1
Hard to take a bath or shower	LEARNS (n=11)	0	0	0	9	91
	Norm (n=964)	0.4	1.2	0.8	2.3	95.2
Hard to do chores around the house	LEARNS (n=11)	0	0	27	9	64
	Norm (n=964)	0.6	2	5.5	8.5	83.4
Hurt or ache	LEARNS (n=11)	9	9	27	45	9
	Norm (n=964)	0.9	4.1	12.3	20.7	61.8
Low energy	LEARNS (n=11)	9	18	36	18	18
	Norm (n=964)	1.2	2.6	10.4	21.2	64.6
Emotional functioning						
Feel afraid or scared	LEARNS (n=11)	0	0	18	27	55
	Norm (n=964)	0.3	3.2	11.6	29.8	55.1
Feel sad or blue	LEARNS (n=11)	0	0	45	27	27
	Norm (n=964)	0.7	4.1	20.4	34.8	39.9
Feel angry	LEARNS (n=11)	9	18	27	27	18
	Norm (n=964)	0.4	4.3	26.1	33.6	35.6
Trouble sleeping	LEARNS (n=11)	9	9	45	9	27
	Norm (n=964)	2.1	6.7	17	27.6	46.6
Worry about what will happen	LEARNS (n=11)	36	9	9	18	27
	Norm (n=964)	1.3	4.6	11.5	27.5	55.1
Social functioning						
Trouble getting along with peers	LEARNS (n=11)	0	0	9	18	73
	Norm (n=964)	0.4	3.3	12.8	33.7	49.8
Other kids not wanting to be friends	LEARNS (n=11)	0	0	0	27	73
	Norm (n=964)	1.1	5.3	14.5	29.3	49.8
Teased	LEARNS (n=11)	0	0	9	0	91
	Norm (n=964)	0.5	1.8	11.9	22.9	62.9
Doing thing other peers do	LEARNS (n=11)	0	0	0	55	45
	Norm (n=964)	1.1	3.3	14	23	58.5
Hard to keep up when play with others	LEARNS (n=11)	0	0	0	55	45
	Norm (n=964)	0.5	2.6	7.7	18.7	70.5
School functioning						
Focus	LEARNS (n=11)	0	0	9	45	45
	Norm (n=964)	2.1	6.2	27.2	27.6	36.9
Forgetting things	LEARNS (n=11)	0	0	27	36	36
	Norm (n=964)	1	7.2	25.9	32.5	33.4
Trouble keeping up with schoolwork	LEARNS (n=11)	0	18	0	36	45
	Norm (n=964)	2.2	6.4	17.4	26.3	47.6
Miss school – not well	LEARNS (n=11)	0	9	9	45	36
	Norm (n=964)	0.3	0.9	7.7	21.8	69.3
Miss school – doctor appointment	LEARNS (n=11)	9	0	36	36	18
	Norm (n=964)	0	1.2	9	21.4	68.4

Table S2 Behavioral and emotional difficulties scores of the parent-reported (P) and self-reported versions (C) of the SDQ. Cohen's *d* indicates the effect size. Effect sizes of 0.2, 0.5, and 0.8 were considered small, moderate, and high, respectively. Higher scores on Prosocial behavior reflect strengths, higher scores on the four negative attributions reflect difficulties. Total difficulties is the sum of negative attribution scales (range 0-40), Internalizing problems the sum of the Emotional symptoms and Conduct problems scales (range 0-20), and Externalizing problems the sum of the Hyperactivity-inattention and Peer problems scales (range 0-20). - = no scores available due to low internal inconsistency (α < 0.500); SD = standard deviation; SDQ = Strengths and Difficulties Questionnaire

	SDQ scores						*Clinical scores*	
	**SSNS N=7**		**Reference[a] N=194**				**SSNS**	**Reference**
**2-3 years (P)**					***P*[c]**			
Age, mean (SD)	3.2 (0.4)		3.0 (0.6)		0.35			
Male, n (%)	4 (57)		100 (51.5)					
**SDQ subscales**	**Mean**	**SD**	**Mean**	**SD**	***P*[c]**	***d* (95% CI)**	**n (%)**	**n (%)**
Emotional symptoms	2.9	2.3	-	-	-	-	-	-
Conduct problems	1.4	1.4	-	-	-	-	-	-
Hyperactivity-inattention	3.4	3.4	3.7	2.2	0.82	-0.13 (-0.89-0.62)	1 (14.3%)	21 (10.8)
Peer problems	2.3	1.8	-	-	-	-	-	-
Prosocial behavior	6.4	2.1	7.6	1.7	0.19	-0.70 (-1.46-0.06)	3 (42.9%)	16 (8.2)
Total difficulties	10.0	6.1	7.8	4.0	0.38	0.54 (-0.21-1.29)	1 (14.3%)	20 (10.3)
Internalizing problems	5.1	2.5	-	-	-	-	-	-
Externalizing problems	4.9	4.5	5.5	3.0	0.71	-0.20 (-0.95-0.56)	1 (14.3%)	15 (7.7)
	**SSNS N=11**		**Reference[a] N=182**				**SSNS**	**Reference**
**4-5 years (P)**					***P*[c]**			
Age, mean (SD)	4.8 (0.6)		5.0 (0.6)		0.27			
Male, n (%)	7 (64)		112 (61.5)					
**SDQ subscales**	**Mean**	**SD**	**Mean**	**SD**	***P*[c]**	***d* (95% CI)**	**n (%)**	**n (%)**
Emotional symptoms	1.5	1.5	1.3	1.6	0.66	0.13 (-0.48-0.73)	2 (18.2%)	15 (8.2)
Conduct problems	0.9	0.7	-	-	-	-	-	-
Hyperactivity-inattention	2.4	2.5	3.2	2.5	0.30	-0.32 (-0.93-0.28)	1 (9.1%)	23 (12.6)
Peer problems	1.0	1.4	-	-	-	-	-	-
Prosocial behavior	8.0	1.9	8.1	1.9	0.91	-0.05 (-0.66-0.56)	2 (18.2%)	17 (9.3)
Total difficulties	5.7	3.1	6.7	4.8	0.32	-0.21 (-0.82-0.40)	0 (0%)	17 (9.3)
Internalizing problems	2.5	2.2	2.2	2.4	0.75	0.13 (-0.48-0.73)	1 (9.1%)	14 (7.7)
Externalizing problems	3.3	2.7	4.5	3.3	0.17	-0.37 (-0.98-0.24)	1 (9.1%)	15 (8.2)
	**SSNS N=17**		**Reference[a] N=403**					
**6-11 (P)**					***P*[c]**			
Age, mean (SD)	8.6 (1.9)		8.6 (1.7)					
Male, n (%)	12 (71)		202 (50.1)					

SDQ subscales	Mean	SD	Mean	SD	*P*[c]	*d* (95% CI)	n (%)	n (%)
Emotional symptoms	1.9	1.8	2.1	2.2	0.65	-0.09 (-0.58-0.39)	2 (11.8%)	30 (7.4)
Conduct problems	0.9	0.7	1.2	1.4	0.13	-0.22 (-0.70-0.27)	0 (0%)	29 (7.2)
Hyperactivity-inattention	2.7	2.1	3.6	2.8	0.10	-0.32 (-0.81-0.16)	0 (0%)	48 (11.9)
Peer problems	0.5	0.5	1.3	1.8	**<0.001**	-0.45 (-0.94-0.03)	0 (0%)	47 (11.7)
Prosocial behavior	9.2	1.0	8.3	1.9	**0.001**	0.48 (0.00-0.97)	0 (0%)	38 (9.4)
Total difficulties	5.9	3.6	8.1	6.2	**0.022**	-0.36 (-0.85-0.13)	0 (0%)	41 (10.2)
Internalizing problems	2.4	2.4	3.4	3.4	0.053	-0.30 (-0.78-0.19)	0 (0%)	34 (8.3)
Externalizing problems	3.6	2.6	4.8	3.7	0.08	-0.33 (-0.81-0.16)	0 (0%)	40 (9.9)
	**SSNS N=4**		**Reference[b] N=736**					
**12-17 (P)**					***P*[c]**			
Age, mean (SD)	14.6 (0.7)		14.3 (1.4)		0.47			
Male, n (%)	2 (50)		345 (46.9)					
**SDQ subscales**	**Mean**	**SD**	**Mean**	**SD**	***P*[c]**	***d* (95% CI)**	**n (%)**	**n (%)**
Emotional symptoms	3.8	1.0	1.9	2.1	**0.031**	**0.91 (0.10-1.71)**	1 (25.0%)	84 (11.4)
Conduct problems	1.0	0.8	1.0	1.4	>0.99	0.00 (-0.80-0.80)	0 (0%)	84 (11.4)
Hyperactivity-inattention	2.0	0.8	2.7	2.5	0.18	-0.28 (-1.08-0.52)	0 (0%)	72 (9.8)
Peer problems	1.3	1.9	1.5	1.8	0.81	10.11 (-0.91-0.70)	1 (25.0%)	57 (7.7)
Prosocial behavior	9.8	0.5	8.3	1.8	**0.010**	**0.84 (0.03-1.64)**	0 (0%)	116 (15.8)
Total difficulties	8.0	3.4	7.0	5.6	0.59	0.17 (-0.62-0.98)	0 (0%)	76 (10.3)
Internalizing problems	5.0	2.7	3.4	3.3	0.32	0.49 (-0.32-1.29)	1 (25.0%)	80 (10.9)
Externalizing problems	3.0	0.8	3.7	3.3	0.18	-0.21 (-1.02-0.59)	0 (0%)	65 (8.8)
	**SSNS N=4**		**Reference[b] N=993**					
**12-17 years (C)**					***P*[c]**			
Age, mean (SD)	13.7 (1.6)		14.4 (1.5)					
Male, n (%)	3 (50)		466 (46.9)					
**SDQ subscales**	**Mean**	**SD**	**Mean**	**SD**	***P*[c]**	***d* (95% CI)**	**n (%)**	**n (%)[d]**
Emotional symptoms	2.5	1.1	2.3	2.1	0.66	0.10 (-0.89-1.07)	0 (0%)	--
Conduct problems	0.8	1.0	1.3	1.3	0.30	-0.38 (-1.37-0.60)	0 (0%)	--
Hyperactivity-inattention	3.5	1.1	3.7	2.4	0.66	-0.08 (-1.07-0.90)	0 (0%)	--
Peer problems	1.7	1.4	1.4	1.6	0.65	0.19 (-0.79-1.16)	1 (16.7%)	--
Prosocial behavior	9.3	0.5	8.1	1.6	**0.002**	0.75 (-0.23-1.73)	0 (0%)	--
Total difficulties	8.5	3.1	8.6	5.0	0.94	-0.02 (-1.00-0.96)	0 (0%)	--
Internalizing problems	4.2	1.7	3.6	3.1	0.46	0.19 (-0.79-1.18)	0 (0%)	--
Externalizing problems	4.3	1.8	5.0	3.1	0.39	-0.23 (-1.21-0.76)	0 (0%)	--

[a] Reference data from Maurice-Stam et al. (2018).
[b] Reference data from Vugteveen et al. (2021).
[c] *P* values were calculated using a one-sample t test.
[d] Proportions of reference children aged 12-17 (self-report version) were not available (Vugteveen et al., 2021).

Table S3 Results of the univariate regression analysis (after adjusting for multicollinearity) to identify clinical variables that are associated with the outcomes of **a.** the Pediatric Quality of Life Inventory 4.0 (PedsQL), both total HRQoL and subdomain scores, and **b.** the Strength and Difficulties Questionnaire (SDQ). Standardized regression coefficients (β) and corresponding *P* values are shown. Clinical variables that had a significance level of < 0.05 (shown in bold) were included in the final multiple regression analysis model. HRQoL = Health-related quality of life, NL/BE = born in the Netherlands or Belgium

**a. PedsQL**	**Total HRQoL**		**Physical functional**		**Emotional functioning**		**Social functioning**		**School functioning**		**Psychosocial functioning**	
	**β**	***P***	**β**	***P***	**β**	***P***	**β**	***P***	**β**	***P***	**β**	***P***
**Child characteristics**												
Age (years)	-0.30	0.058	-0.30	0.064	-0.34	**0.034**	0.08	0.64	-0.31	0.053	-0.28	0.09
Sex (male)	0.09	0.57	0.11	0.51	0.20	0.21	-0.04	0.81	-0.08	0.64	0.08	0.61
**Disease characteristics**												
Time-to-remission (days)	-0.17	0.30	-0.15	0.35	-0.32	**0.045**	0.03	0.83	0.04	0.79	-0.17	0.31
Illness (days)	-0.08	0.63	-0.15	0.34	-0.07	0.67	0.08	0.62	-0.01	0.96	-0.02	0.92
School absence (days)	-0.03	0.88	-0.10	0.53	-0.04	0.79	0.17	0.29	0.00	0.98	0.03	0.83
Steroid side-effects present (yes)	-0.14	0.40	-0.16	0.31	-0.12	0.47	-0.11	0.49	0.02	0.88	-0.10	0.55
Moon face	-0.11	0.49	-0.13	0.43	-0.07	0.67	-0.07	0.66	-0.05	0.75	-0.09	0.58
Mood changes	0.04	0.82	0.03	0.83	-0.05	0.75	0.16	0.33	0.07	0.68	0.04	0.82
Behavioral problems	0.07	0.65	0.15	0.34	-0.08	0.61	0.04	0.83	0.13	0.44	0.00	0.99
Weight gain	0.05	0.78	0.13	0.43	0.02	0.92	-0.11	0.49	-0.01	0.96	-0.02	0.91
Binge eating	-0.14	0.38	-0.17	0.30	-0.04	0.79	-0.02	0.92	-0.16	0.32	-0.11	0.51
Medication used (n)	-0.12	0.47	-0.16	0.33	0.07	0.69	0.06	0.69	-0.30	0.064	-0.08	0.63
**Sociodemographics parents**												
Country of birth (NL/BE)	0.09	0.60	0.13	0.44	0.06	0.73	-0.14	0.39	0.16	0.33	0.05	0.77
Educational level (high)	0.21	0.58	0.11	0.76	0.26	0.48	0.03	0.94	0.24	0.52	0.25	0.51

b. SDQ	Emotional symptoms		Conduct problems		Hyperactivity-Inattention		Peer problems		Prosocial behavior		Total difficulties		Internalizing problems		Externalizing problems	
	**β**	***P***	**β**	***P***	**β**	***P***	**β**	***P***	**β**	***P***	**β**	***P***	**β**	***P***	**β**	***P***
**Child characteristics**																
Age (years)	0.19	0.25	-0.09	0.59	-0.10	0.56	-0.24	0.14	0.51	**<.001**	-0.07	0.67	0.01	0.97	-0.11	0.52
Sex (male)	-0.01	0.97	0.27	0.10	-0.05	0.78	0.33	**0.039**	-0.19	0.25	0.14	0.41	0.18	0.28	0.04	0.79
**Disease characteristics**																
Time to remission (days)	0.06	0.72	0.10	0.53	0.08	0.64	-0.16	0.33	0.06	0.73	0.04	0.81	-0.05	0.78	0.09	0.57
Illness (days)	0.00	0.98	-0.11	0.52	-0.01	0.94	-0.14	0.40	0.15	0.36	-0.08	0.64	-0.08	0.63	-0.04	0.80
School absence (days)	0.00	0.99	-0.27	0.09	-0.08	0.64	-0.20	0.23	0.38	**0.016**	-0.16	0.32	-0.11	0.50	-0.14	0.38
Steroid side-effects present (yes)	-0.13	0.44	0.18	0.28	0.15	0.37	0.01	0.96	-0.06	0.71	0.07	0.68	-0.09	0.59	0.17	0.29
Moon face	0.03	0.84	0.03	0.87	0.06	0.70	-0.10	0.55	-0.03	0.86	0.02	0.88	-0.03	0.86	0.06	0.72
Mood changes	-0.04	0.82	0.03	0.85	0.19	0.26	-0.02	0.92	-0.18	0.26	0.09	0.59	-0.04	0.82	0.16	0.32
Behavioral problems	-0.06	0.72	0.28	0.08	0.35	**0.028**	-0.13	0.42	0.04	0.80	0.19	0.25	-0.12	0.48	0.37	**0.020**
Weight gain	-0.20	0.22	-0.02	0.90	0.08	0.63	-0.01	0.94	-0.03	0.86	-0.05	0.75	-0.16	0.34	0.06	0.72
Binge eating	-0.33	**0.039**	0.02	0.89	0.14	0.41	0.05	0.74	-0.04	0.82	-0.05	0.78	-0.22	0.19	0.12	0.47
Medication used (n)	0.07	0.66	0.15	0.35	0.14	0.41	0.12	0.48	-0.11	0.52	0.18	0.28	0.12	0.47	0.16	0.34
**Sociodemographics parents**																
Country of birth (NL/BE)	-0.04	0.80	0.01	0.93	0.19	0.26	0.16	0.34	-0.16	0.35	0.14	0.39	0.06	0.73	0.16	0.34
Educational level (high)	-0.38	0.10	0.00	>0.99	0.05	0.84	0.06	0.81	-0.19	0.38	-0.12	0.62	-0.25	0.27	0.04	0.87
